# The Vagus Nerve as a Gateway to Body Ownership: taVNS Reduces Susceptibility to a Virtual Version of the Cardiac and Tactile Rubber Hand Illusion

**DOI:** 10.1111/psyp.70040

**Published:** 2025-03-17

**Authors:** Alisha Vabba, Keisuke Suzuki, Milica Doric, Tim J. Möller, Sarah Garfinkel, Hugo Critchley

**Affiliations:** ^1^ Department of Clinical Neuroscience, Brighton and Sussex Medical School University of Sussex Brighton UK; ^2^ Sapienza University of Rome and CLN2S@Sapienza Istituto Italiano di Tecnologia Roma Italy; ^3^ Center for Human Nature, Artificial Intelligence and Neuroscience (CHAIN) Hokkaido University Sapporo Japan; ^4^ Department of Clinical and Movement Neurosciences University College London London UK; ^5^ Touro University Berlin Berlin Germany; ^6^ Institute of Cognitive Neuroscience University College London London UK

## Abstract

Transcutaneous auricular vagus nerve stimulation (taVNS) has been shown to influence cognitive and emotional function and enhance interoceptive awareness. This study investigates if taVNS effects extend to the experience of body ownership, as measured via susceptibility to the rubber hand illusion (RHI) in a virtual reality setting. The experiment involved 27 participants who underwent real and sham stimulation in two separate sessions while experiencing synchronous or asynchronous visuo‐cardiac and visuo‐tactile feedback on a virtual arm in place of their own. Results indicated that active compared to sham taVNS decreased sensitivity to the illusion in both cardiac and tactile trials. Specifically, a greater proprioceptive drift difference (PDD) toward the rubber hand was observed for synchronous compared to asynchronous trials only during sham (*t*(26) = −4.58, *p*_bonf_ < 0.001) but not during active (*p*_bonf_ = 1.00) stimulation. A similar pattern was also observed for subjective ownership, where synchronous trials led to greater subjective ownership than asynchronous trials only during sham (*t*(26) = −3.52, *p*_bonf_ = 0.010) but not during active (*p*_bonf_ = 1.00) stimulation. These findings suggest that stimulation might enhance body ownership, making individuals more attuned to their real bodily signals and less susceptible to bodily illusions. Additionally, physiological measures such as heart rate (HR), heart rate variability (HRV), and skin sympathetic nervous activity (SKNA) were assessed to explore the autonomic effects of taVNS. We observed a decrease in HR during active stimulation (*t*(26) = 4.30, *p*_bonf_ < 0.001), and an increase in SKNA during both sham (*t*(26) = −4.40, *p*_bonf_ < 0.001) and active stimulation (*t*(26) = −4.85, *p*_bonf_ < 0.002). These findings contribute to the understanding of the vagus nerve's role in integrating visceral and somatosensory signals, with implications for clinical applications in conditions characterized by altered interoception and body ownership.

## Impact Statement

This study provides novel evidence that transcutaneous auricular vagus nerve stimulation (taVNS) reduces susceptibility to the rubber hand illusion (RHI), suggesting a role for the vagus nerve in body ownership and multisensory integration.

## 1 Introduction

Afferent fibers innervate almost all tissues of the body, and information about the physiological state of the body reaches the brain via cranial (e.g., the vagal and glossopharyngeal nerves), spinal (lamina‐I spinal‐thalamocortical) and humoral pathways (chemicals carried in the blood such as glucose, cortisol, insulin). One of the channels of this body‐to‐brain communication is the vagus nerve, which carries signals from many organs to converge in the nucleus tractus solitarius (NTS) in the brainstem, where they are relayed to brainstem nuclei for homeostatic control and, via thalamocortical projections, to higher order structures such as the thalamus, hippocampus, amygdala, and insula (Craig 2002; Critchley and Harrison 2013). In the insular and somatosensory cortices, interoceptive and exteroceptive signals are integrated into a cortical representation of the state of the body, giving rise to bodily sensations (Craig 2009).

The past decades have shown a rising interest in interoception—the neural and mental representation of the physiological state of the body—and its relevance to bodily self‐representation as well as higher order cognitive, emotional, and social function (Critchley and Garfinkel 2017; Feldman et al. 2024). Interoception is considered a multi‐dimensional hierarchical construct comprising different levels, from the preconscious effects of low‐level visceral afferents on cognition and behavior (e.g., cognition at different stages of the cardiac cycle) to the conscious processing of physiological signals, such as the capacity to accurately detect signals, known as interoceptive accuracy, the self‐reported tendency to pay attention to these signals, known as interoceptive sensibility, and the meta‐cognitive awareness of ones’ capacity to detect signals, termed interoceptive awareness (Garfinkel et al. 2015).

Interoception plays a fundamental role in contributing to our sense of self (Critchley and Garfinkel 2018; Seth 2013). For example, experiments show that signals from the heart can affect the experience of body ownership, the feeling that one's body parts belong to oneself, forming a crucial aspect of self‐awareness (Longo et al. 2008; Tsakiris 2010). Early studies suggested that higher interoceptive accuracy (e.g., better heartbeat detection) is associated with reduced susceptibility to the rubber hand illusion (RHI), possibly because individuals with heightened interoception rely more on their internal bodily signals rather than external visual and tactile cues (Tsakiris et al. 2011). However, this correlation has been debated, as several studies have failed to replicate the effect (Crucianelli et al. 2018; Horváth et al. 2020).

These findings suggest that interoceptive sensitivity alone is not sufficient to determine susceptibility to bodily illusions like the RHI, which arise from the complex interaction between interoceptive, proprioceptive, and exteroceptive signal integration processes. However, studies show that disrupting multisensory integration by enhancing interoceptive cues can affect the illusion. In virtual versions of the task, if the rubber hand (or full body) flashes in time with the person's heartbeat, this can enhance the illusion (Aspell et al. 2013; Suzuki et al. 2013). One proposed explanation is that individuals might experience cardiac feedback from the fingers (in the case of the rubber hand) or from the chest (in the case of the full body), and if these sensations match the visual stimuli on the hand or whole body, the brain may integrate them as a common sensory event, reinforcing the illusion of ownership.

Finally, some studies suggest that the link between interoceptive awareness and body ownership may be bidirectional, as changes in body ownership can also affect interoceptive sensitivity (Filippetti and Tsakiris 2017) and changes in corporeal awareness induced by the full‐body illusion are linked to heartbeat‐evoked potentials (HEP) an electrophysiological brain response attributed to cortical processing of the heartbeat (Heydrich et al. 2018).

Additionally, interoceptive measures are associated with higher order functions such as emotional regulation and reactivity (for recent reviews see Pinna and Edwards 2020; Quadt et al. 2018; Tsakiris and Critchley 2016) and with aspects of social cognition and behavior, such as perspective taking (Shah et al. 2017), empathic response (Ainley et al. 2015; Ernst et al. 2013; Fukushima et al. 2011; Heydrich et al. 2021), altruistic behavior (Piech et al. 2017), and moral decision‐making (Ditto et al. 2006; Lenggenhager et al. 2013; Mancini et al. 2011; Nicolardi et al. 2020; Vabba et al. 2022; Williams et al. 2016). Relatedly, dysfunctional processing of interoceptive signals is observed in neurological and psychiatric disorders, including depression, panic disorder, eating disorders, substance abuse, anxiety disorders, depersonalization, and derealisation disorders (Khalsa et al. 2018).

Emerging perspectives suggest that vagus nerve stimulation (VNS) can influence interoceptive processes. Teckentrup and Kroemer (2024) discuss potential mechanisms through which VNS could modulate interoception, proposing that stimulation may enhance the brain's ability to process internal bodily signals. Given its major role in the afferent communication of visceral information along the body–brain axis, stimulation of the VNS to manipulate interoceptive processing is a promising tool for research into bodily self‐consciousness and for clinical practice, notably in psychiatric disorders associated with perturbation of self‐consciousness (see Khalsa et al. 2018 for a review). Stimulation of the VNS with an implanted device in the chest is an established clinical treatment for refractory epilepsy (Conway et al. 2015), depression (Conway et al. 2015), heart failure (De Ferrari et al. 2011), obesity (Val‐Laillet et al. 2010), and chronic pain (Kirchner et al. 2000). Recently, attention has focused on Arnold's nerve, a small branch of the VNS that innervates part of the ear. Stimulation of this area, non‐invasive transcutaneous auricular vagus nerve stimulation (taVNS), elicits many of the effects of invasive VNS, but safely without the need for surgery. Both procedures activate the same relevant “interoceptive” brain areas (Badran, Dowdle, et al. 2018; Frangos et al. 2015; Kraus et al. 2013) and taVNS is increasingly used as an experimental tool to modulate cognitive and emotional function (Burger et al. 2016; Colzato et al. 2017, 2018; Jongkees et al. 2018; Sellaro et al. 2018). Importantly, a recent study by Villani et al. (2019) found that taVNS improved perceptual performance accuracy in the heartbeat discrimination task (Whitehead et al. 1977), highlighting the potential of taVNS to manipulate interoception. Additionally, neuroimaging work has explored the effects of taVNS on gastric coupling, providing preliminary evidence for the stimulation's impact on interoceptive pathways (Müller et al. 2022).

To consolidate and develop the use of taVNS, it is important to examine its effect on manipulations of the interoceptive system and on higher levels of self‐processing, for example, the experience of body ownership (EBO)—the subjective feeling that one's body and body parts belong to oneself (Paciorek and Skora 2020; Villani et al. 2019). The RHI is a well‐known experimental paradigm used to investigate body ownership. In the illusion, participants' real hand is hidden while a rubber hand is placed in front of them. When the rubber hand is stroked synchronously with their real hand, participants often experience the rubber hand as their own (Botvinick and Cohen 1998). This phenomenon arises from the multisensory integration of visual, tactile, and proprioceptive signals, with the brain resolving conflicting sensory inputs to update the perceived location and ownership of the limb (Ehrsson et al. 2004; Ehrsson 2020; Kilteni et al. 2015). This multisensory integration involves neural mechanisms in the premotor cortex, intraparietal sulcus, and insula, which contribute to a coherent sense of the body in space (Ehrsson et al. 2004). Recent studies suggest that when proprioceptive signals are less reliable, the brain more readily integrates visual and tactile information to infer limb ownership (Chancel et al. 2022; Chancel and Ehrsson 2023).

Given that taVNS has been shown to modulate interoceptive awareness (Villani et al. 2019), this study investigates whether taVNS might influence the multisensory integration underlying body ownership, potentially making individuals less susceptible to the RHI. We test whether sham‐controlled taVNS affects participants' EBO in a virtual reality version of the RHI (Botvinick and Cohen 1998). Participants paid attention to the virtual hand while either (1) synchronous or asynchronous visuo‐cardiac feedback was projected onto it, or (2) a rendered paintbrush stroked the virtual hand synchronously or asynchronously with the experimenter stroking the participants' real hand with a real paintbrush.

In line with evidence that people with stronger cardiac interoception may be less prone toward the illusion (Tsakiris et al. 2011) and that more reliable proprioceptive signals might interfere with the easy integration of visuo‐tactile signals that lead to inferring limb ownership (Chancel et al. 2022; Chancel and Ehrsson 2023), we predicted that active taVNS (compared to sham) would increase interoception and make participants more attuned to their real body signals, making them less susceptible to the illusion in both cardiac and tactile trials. We also investigated whether, in cardiac trials specifically, the stimulation has a differential impact on susceptibility to the illusion based on the synchronicity of the trial. In a previous version of the task (Suzuki et al. 2013), if the simulated hand flashed in time with the participants' heartbeat this increased the illusion compared to asynchronous trials. Thus, we predicted that while in asynchronous trials the stimulation would decrease the strength of the illusion, in synchronous trials, the stimulation might increase susceptibility to the illusion, in accord with the previous version of the task and considering the congruency between the (strengthened) body signal and the visual feedback.

A secondary objective of this study was to examine if any effect of sham‐controlled taVNS on the rubber hand illusion may be related to (1) how perceptually sensitive people are to their own heartbeats, as assessed by behavioral tests of baseline cardiac interoception, and (2) stimulation‐induced changes in autonomic arousal, namely skin sympathetic nervous activity (SKNA), heart rate (HR), and heart rate variability (HRV). Indeed, physiological indices including heart rate and blood pressure are reportedly modulated by taVNS in some (Antonino et al. 2017; Badran, Mithoefer, et al. 2018; Clancy et al. 2014) but not all studies (Burger et al. 2016; Colzato et al. 2017; Villani et al. 2019). Additionally, recent evidence suggests inconsistencies in findings that taVNS alters HRV in humans (Burger et al. 2020; Kaniusas et al. 2019; Wolf et al. 2021).

## 2 Materials and Methods

### 2.1 Participants

A power analysis (*power* = 0.8; *α* = 0.05) using G*Power for an ANOVA with two factors with two levels, and based on the effect size (Cohen's *d* = 0.59) of the proprioceptive drift data of the Suzuki et al. (2013) study, indicated a sample size of 26 participants. A total of 27 participants (13 males, age [*mean* (*M*) = 22.17, *standard deviation* (SD) = 4.45]) recruited via flyers and social media platforms took part in the study. All participants provided written and informed consent, and ethical approval was granted by the ethics committee at the Brighton and Sussex Medical School. We administered a screening form to assess eligibility to undergo taVNS, following previously published protocols. We tested only healthy, non‐pregnant volunteers aged between 18 and 30 years who had no history of neurological or psychiatric disorders, substance abuse, brain surgery, tumors, chronic or acute medication use, susceptibility to seizures or migraine, pacemakers, or other implanted devices. All participants were naïve to taVNS and received no information about the stimulation type or experimental hypotheses.

### 2.2 Procedure

Participants took part in two experimental sessions (real vs. sham taVNS in counterbalanced order) separated by at least 1 week, lasting a maximum of 2 h, including setup and performance in the experimental tasks. During the first session, when participants arrived in the laboratory, they were instructed to read the experimental protocol and sign the consent sheets. They then completed the cardiac interoception tasks (only during the first session). During both sessions, participants were fitted with the neuECG recording and taVNS stimulation setup (see below for details). A 10‐minute recording of physiological activity was conducted, with 5 min at rest and 5 min during taVNS (with electrodes placed on the cymba concha or earlobe based on whether it was a real or sham stimulation session). Once the baseline physiological recording was completed, participants were fitted with an Electrocardiograph (ECG; specifically, the Arduino based mobile ECG (ArdMob‐ECG)) and the head‐mounted display (HMD) before they began the RHI task, which they completed while receiving real or sham taVNS.

### 2.3 Measuring Individual Differences in Cardiac Interoception

To measure individual differences in cardiac interoceptive accuracy, participants completed the heartbeat counting task (HCT; Schandry 1981) and the heartbeat detection task (HDT; Whitehead et al. 1977). During the HCT, participants were instructed to mentally count their heartbeats without using physical cues (e.g., taking their pulse) in six intervals of 25, 30, 35, 40, 45, and 50 s, presented in randomized order. Two auditory tones through a headset delimited time intervals. After each trial, participants reported the number of felt heartbeats with a keyboard and were asked not to estimate the number of heartbeats but to only count heartbeats they truly perceived. During the HDT, participants listened to sequences of 10 auditory tones that were either synchronous (200 ms after the R wave) or asynchronous (500 ms after the R wave) with their real heartbeats, and had to judge the perceived synchronicity. The overall procedure consisted of 20 trials (10 synchronous and 10 asynchronous) presented in pseudo‐randomized order. During interoception tasks, real heartbeats were recorded with a pulse oximeter (Nonin 8600 with a soft sensory fitting to reduce exteroceptive feedback) attached to the ring finger of their left hand.

### 2.4 Transcutaneous Auricular Vagus Nerve Stimulation

**FIGURE 1:**
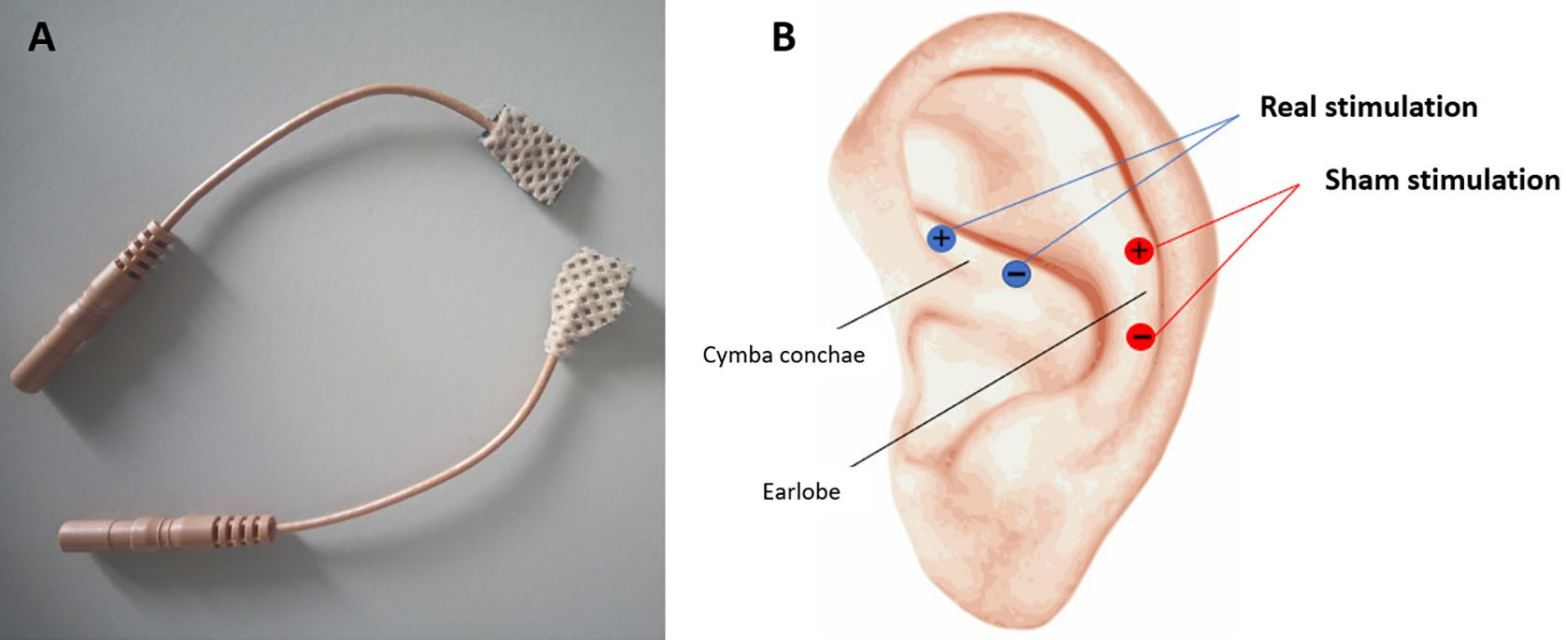
taVNS setup. Panel A:  neurostimulation electrodes were cut into shape to fit participants' ears. Panel B: Electrodes were placed on the cymba conchae during active taVNS (blue) and on the earlobe during sham taVNS (red). We delivered continuous current stimulation with a pulse width of 200–300 μs at 25–30 Hz with stimulation amplitude tailored to individual perceptual threshold (between 0.5 and 50 μA).

We employed a V‐TENS Plus (*Body Health Care ltd*) electrical stimulator to deliver the stimulation to the auricular branch of the vagus nerve. The device was attached to neurostimulation tens electrodes (*HealthcareWorld*) which were originally 32 mm wide and were custom cut into shape to fit the participants' ear (see Figure 1 Panel A).

During active stimulation, the electrodes were placed on the cymba conchae of the left ear, whereas during sham stimulation they were placed on the earlobe, which is not innervated by the VNS (see Figure 1 Panel B). The left ear was used to avoid cardiac effects that have been related to efferent vagal fibers of the right ear. Stimulus parameters followed previously published protocols (Clancy et al. 2014). Specifically, we delivered continuous current stimulation with a pulse width of 200–300 μs at 25–30 Hz with stimulation amplitude tailored to individual perceptual threshold (between 0.5 and 50 μA), which was achieved by slowly increasing the amplitude until the participant reported some sensations (e.g., tingling), which did not cause pain or discomfort. The stimulation amplitude for sham and active stimulation was calibrated separately as the two stimulations occurred on separate days. Following the stimulation, participants answered questions assessing discomfort and pain during the procedure (See Supporting Information).

### 2.5 Measuring Stimulation‐Induced Changes in Autonomic Activity

**FIGURE 2:**
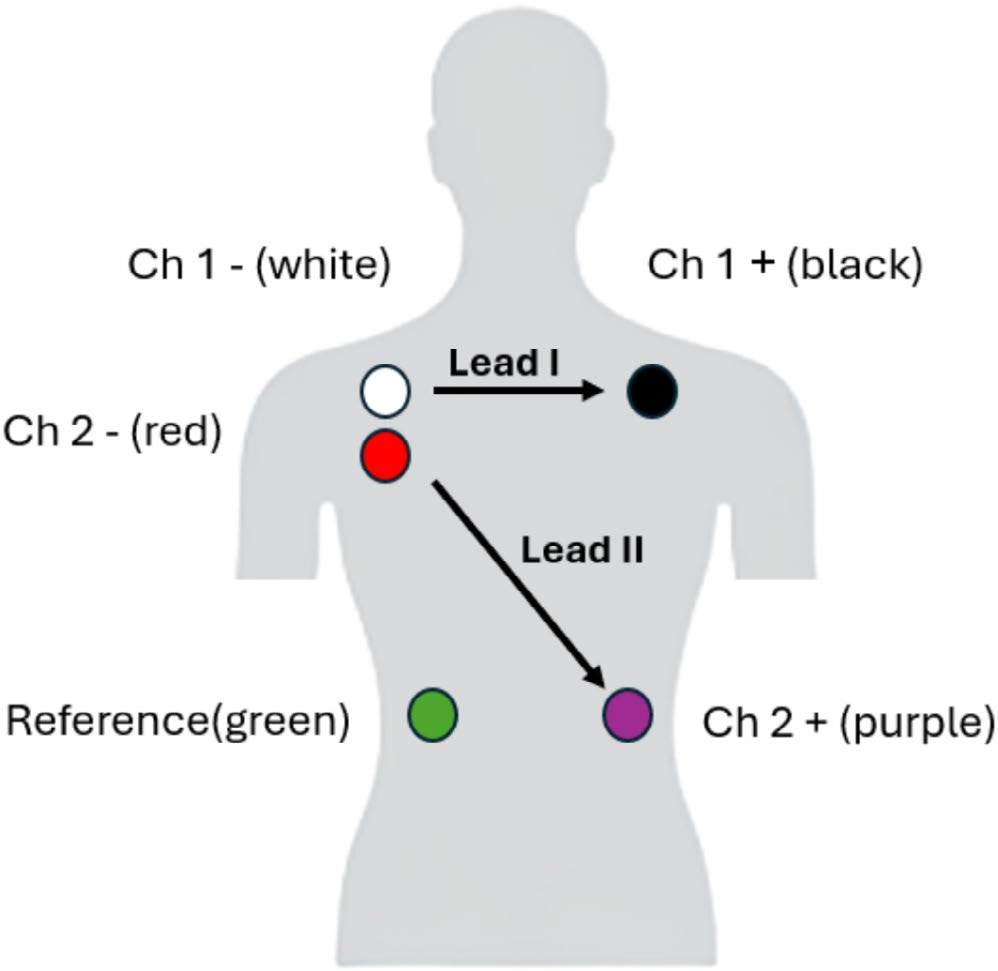
Electrode setup for neuECG recording. The setup was conducted according to the procedures described by Kusayama et al. (2020). Lead I on the right and left subclavicular areas (cathode and anode, respectively) for Channel 1, and Lead II on the right subclavicular area (cathode) and left abdomen (anode) for Channel 2. The reference electrode is placed on the right abdomen.

To analyze whether taVNS induced changes in autonomic function, we measured autonomic activity during 5‐min recordings of baseline (no stimulation), active, and sham taVNS, using a recent method for simultaneously recording the ECG and SKNA (Kusayama et al. 2020). Since the content of these signals falls into different frequency ranges, they can be recorded simultaneously by patch electrodes on the chest (as in traditional ECG), while band‐pass filtering is used to distinguish signals from different sources. Thus, neuECG provides an innovative method to measure sympathetic nerve activity and tone at more fine‐grained detail similar to microneurography, but without the invasive procedures of direct nerve recording. Participants with chest hair were asked to shave prior to the experiment. The skin was cleaned with a preparation gel, dried, and electrodes were placed according to the Einthoven triangle, following the protocol described by Kusayama et al. (2020) which is depicted in Figure 2.

Data was recorded using ADInstrument devices (BioAmp and Powerlab amplifiers) and managed by LabChart Pro 8 software (ADInstruments). Following the protocol, we recorded at a sampling rate of 10.000 Hz. We set the input to ±5 mV for recording ECG signals, with a resolution of 0.1 μV. The data were saved as a LabChart file (.adicht).

### 2.6 The (Virtual) Rubber Hand Illusion

**FIGURE 3:**
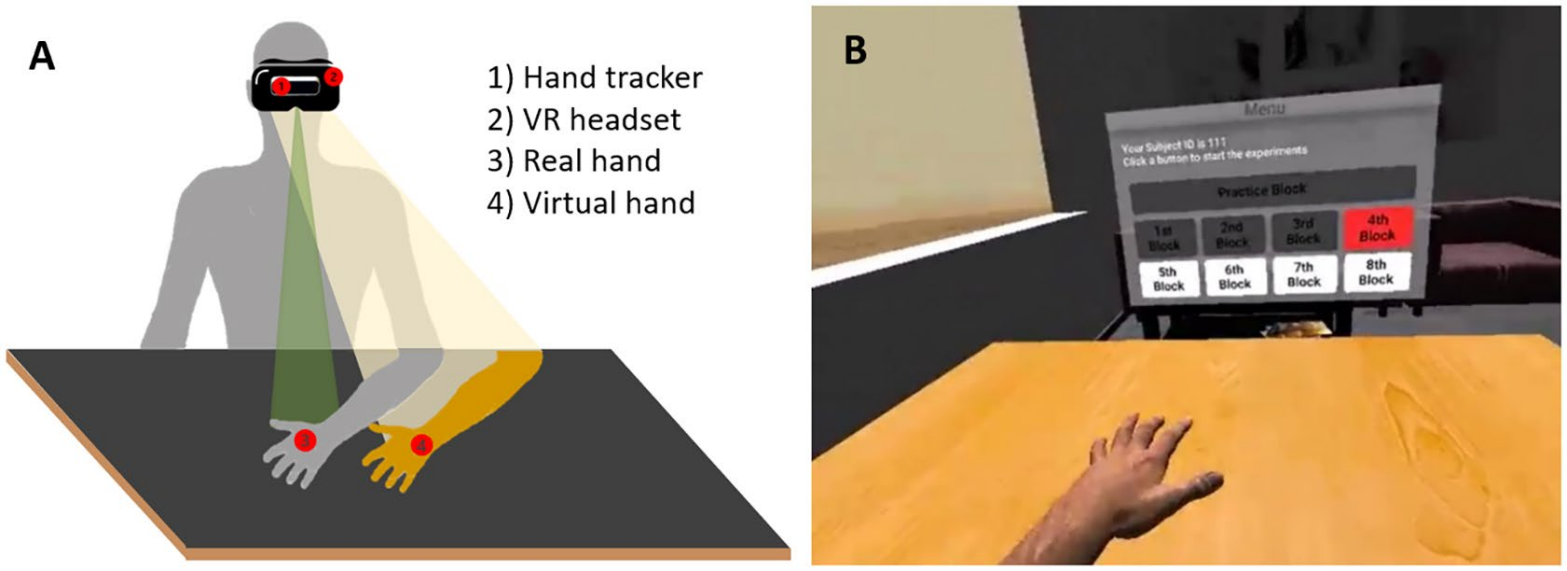
Setup and procedure for the virtual rubber hand illusion. Panel A: Participants sit at a black desk with their left hand on the table. They wear an Oculus Rift head‐mounted display with a LeapMotion hand tracking device on its front. Panel B: In the VR setting, participants are seated at a desk, and they can see a virtual hand that mirrors their hand movements in real time.

The experimental setup (see Figure 3 Panel A) was based on a study by Suzuki et al. (2013).

**FIGURE 4:**
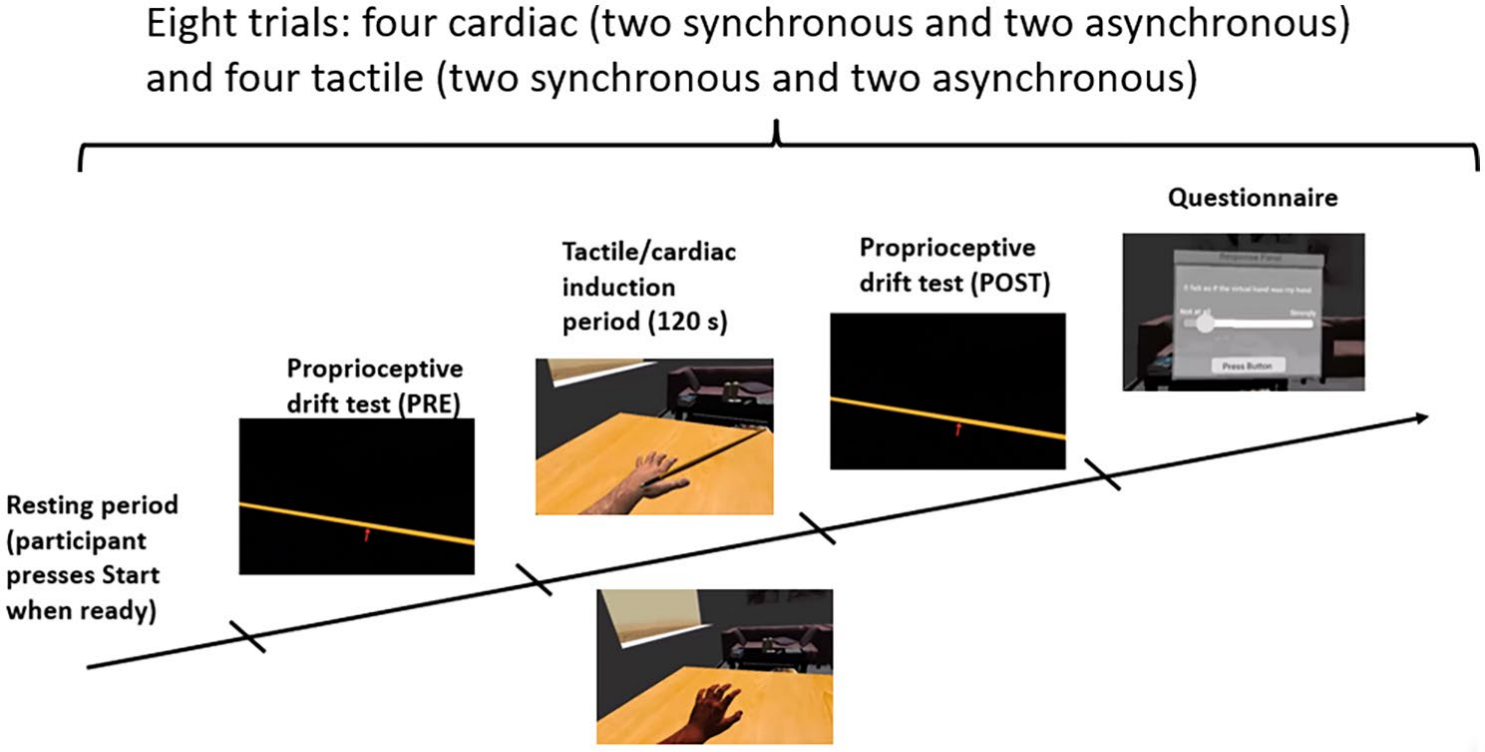
Procedure for the virtual rubber hand illusion. The experiment is composed of eight trials: Four tactile trials (two synchronous and two asynchronous) and four cardiac trials (two synchronous and two asynchronous) which were presented in pseudo‐randomized order. During each trial, after an initial resting period, participants (1) complete a first proprioceptive drift test, (2) focus on their virtual hand during a 2‐min induction period in which the hand is stroked by a paintbrush or flashes red, (3) complete a second proprioceptive drift test, and (4) complete the final 5‐item questionnaire.

Participants were seated at a rectangular desk with their left hand on the table in front of them. While the original study used a pseudo‐3D augmented reality hand captured by a 3D depth camera, here we used a hand tracking device (Leap Motion Controller by Ultraleap) fitted to the front of the HMD participants wore (Oculus Rift CV1) which animated a CGI‐based virtual hand. The desk was covered by black cardboard to facilitate infrared hand tracking. In the virtual reality setting (see Figure 3 Panel B) participants were seated at a virtual desk located in a neutral living room setting. A three‐dimensional hand model with physiognomic characteristics set according to the participant (male/female and light/medium/dark skin tone) was matched via the LeapMotion software (V4 Orion, https://blog.leapmotion.com/v4/) to the participants’ real hand, replicating their movements in near real time. At the start of the experiment and during inter‐trial intervals, participants could freely move their hand, whereas during the tactile/cardiac induction period, participants were required to keep their hand still so as not to induce visuomotor incongruence with the stimulation. For a graphical representation of the task procedure, see Figure 4.

The task consisted of eight trials overall: four tactile trials (two synchronous and two asynchronous) and four cardiac trials (two synchronous and two asynchronous), which were presented in pseudo‐randomized order. Before each trial, there was a resting period during which participants were allowed to move their left hand freely (the movement was mirrored in the virtual setting) and position it comfortably. When participants were ready to start each trial, they pressed start and the virtual hand shifted to a position 19 cm to the left compared to their actual left hand, to create a mismatch of felt and seen location. During the trial, the presented virtual hand was immobilized regardless of the participant's actual left‐hand movements. Participants then completed a first proprioceptive drift test (PD, see below for details). This was followed by an embodiment induction period (120 s) during which the participants were instructed to pay attention to their virtual hand (which was either stroked by a paintbrush or flashed red whenever a heartbeat was detected based on condition). After the embodiment induction followed a second PD test and consecutively a short questionnaire on participants' subjective embodiment experience. Details of each stage are described as follows:

1. *Proprioceptive drift*. During this test, participants were presented via the HMD with a black scene containing a virtual ruler and a cursor, and they had to scroll using the mouse to point to the perceived location of their real left hand. PD was calculated as the distance in centimeters between the participants' real hand and the perceived location thereof. The initial position of the cursor on the ruler was pseudo‐randomized for every trial to avoid participants memorizing prior positions (Tsakiris et al. 2011). Proprioceptive drift difference (PDD) was calculated as the difference in PD from before versus after the induction.
2. *Tactile/cardiac embodiment induction period*. Each induction lasted 2 min, which is argued to be sufficient to induce the illusory experience (Suzuki et al. 2013).
  - During *visuo‐tactile trials*, the experimenter stroked the participants' hands in a ∼1 Hz rhythm with a paintbrush attached to the Oculus Controller. A 3D paintbrush was rendered in the VR setting to match the Oculus controller movements adjusted for the 19‐cm hand drift. In synchronous trials, participants felt the brush on their skin while seeing the virtual brush stroke the virtual hand at the same time. In asynchronous trials, the virtual brush moved with a delay of 500 ms, inducing a temporal mismatch of haptic vs. visual feedback.
  - During *cardiac trials*, the ArdMob‐ECG detected participants' R‐peaks and triggered Unity to modify the near real time (for a discussion of near real time and hardware and software delays, see Möller et al. (2024) rendering of the 3D hand model, making it flash red for 300 ms: The color increased linearly in intensity to 40% alpha) and, after 150 ms, decreased linearly again. In synchronous feedback conditions, the cardio‐visual feedback started immediately, whereas during asynchronous feedback conditions, it was delayed by 500 ms, inducing a temporal mismatch between the real heartbeat and visual feedback.
3. *Subjective ratings*. In the short questionnaire presented after each trial, participants responded to five questions presented via the HMD (see Table 1). The first four questions were adapted from the first RHI (Botvinick and Cohen 1998). Two questions (Q1 and Q3) assessed the strength of the subjective experience of hand ownership, two questions (Q2 and Q4) were control measures, and the final question asked participants to judge the synchronicity of the cardiac or tactile feedback. Responses were collected by means of a questionnaire that was displayed in the VR environment, and participants selected responses by scrolling and clicking with a mouse with their right hand on a 7‐point continuous visual analogue Likert scale ranging from 0 = “strongly disagree” to 6 = “strongly agree”.

**TABLE 1:** Sense of ownership and temporal matching questionnaire.

Question	
Q1	It felt as if the virtual hand was my hand
Q2	It seemed as if I had more than one left hand
Q3	It seemed as if I were feeling a table in the location where the virtual hand was
Q4	It felt as if I no longer had a left hand, as if my left hand had disappeared
Q5	Was visual feedback synchronized to the tactile stimuli? [Tactile]/was cardio feedback in time with your own heartbeats? [Tactile]

*Note:* Q1 and Q3 assess subjective ownership of the virtual hand, and Q2 and Q4 are control questions which do not relate specifically to body ownership. Q5 assesses whether subjects can detect synchronous as compared to asynchronous feedback. Responses were collected on a 7‐point continuous visual analogue scale from 0 = “strongly disagree” to 6 = “strongly agree”.

#### 2.6.1 ECG For the Cardiac Rubber Hand Illusion

To record the heartbeat during the cardiac version of the rubber hand illusion, we used an Arduino‐based mobile ECG (ArdMob‐ECG) featuring an Arduino Mega 2560 microcontroller (Arduino, New York, USA) with an AD8232 heart rate monitor (SparkFun Electronics, Colorado, USA), which was attached to three electrodes placed on the participants' chest according to the Einthoven triangle. The ArdMob‐ECG provides an on‐board real‐time analysis of the heartbeat using the Pan‐Tompkins algorithm and directly sends the detected R‐peaks to Unity without the need for further computations to the computer (Möller et al. 2022). The ArdMob‐ECG has been used successfully in a multitude of different studies. An assessment regarding its delay, specificity, sensitivity, and overall performance against other ECGs is reviewed in detail in Möller et al. (2024).

### 2.7 Analytic Plan

#### 2.7.1 Calculating the Scores of the Interoceptive Tasks

Performance in the HCT was calculated as the ratio of perceived vs. recorded heartbeats averaged across all trials, using the following formula, where scores closer to 1 indicate higher performance in the task:

(1) $$\begin{array}{c} \mathrm{HCT}\;\text{score}=\\ ¼\;\sum \;\left(1–\left[\text{recorded heartbeats}-\text{counted heartbeats}\right]/\text{recorded heartbeats}\right) \end{array}$$

Performance in the heartbeat detection task (HDT) was calculated as the ratio of correct trials.

#### 2.7.2 Calculating Physiological Indices of HR, HRV, and SKNA

All physiological data were analyzed using *LabChart Pro 8* software (*ADInstruments*).

**FIGURE 5:**
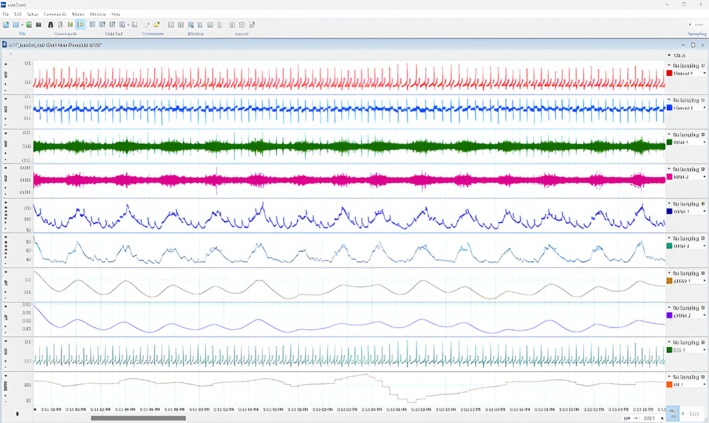
Output data for neuECG from one participant on Labchart 8. Channels 1 and 2 display raw data. Channels 3 and 4 display the SKNA (μV); Channels 5 and 6 display iSKNA; Channels 7 and 8 display aSKNA; Channel 9 displays ECG, and Channel 10 displays the tachogram of the HR.

HR and SKNA were calculated following the analysis protocol described by Kusayama et al. (2020). Figure 5 provides an example of the physiological output for a participant in the study.

Channels 1 and 2 were set to display the raw data for Leads I and II, respectively. Channels 3 and 4 were programmed to display the SKNA (SKNA 1 and 2, respectively) by applying a band‐pass digital filter between 500 and 1000 Hz. Channels 5 and 6 displayed iSKNA1 and iSKNA2, which were the integral absolute values of the respective SKNA, with the time constant decay set to 0.1 s, following standard microneurography protocols. Channels 7 and 8 displayed aSKNA1 and aSKNA2 with a window of 5 s, which were calculated using the arithmetic formula SmoothSec(Abs(Ch3),5) and SmoothSec(Abs(Ch4),5). Channel 9 displayed the ECG with a band‐pass filter of 0.05–150 Hz, and for Channel 10, we used the cyclic measurement setting to show the tachogram of the HR.

RMSSD was calculated as a measure of HRV using the Heart Rate Variability (HRV) 2.0.3. Analysis Software (MLS310/8) add‐on for Labchart. Data were additionally visually inspected for artifacts.

To test whether taVNS induces changes in physiological measures, we used t‐tests to compare the average HR, HRV (via RMSSD), and SKNA during the 5‐min recordings at rest (baseline) and the 5‐min recordings during stimulation (real or sham).

#### 2.7.3 Stimulation Effects of Ownership of the Rubber Hand

For analysis, we used the RStudio 4.4.1. packages readxl, psych, ggplot2, reshape2, dplyr, performance, interactions, lme4, car, jtools, rstatix, afex, ez, emmeans.

PPD was calculated as the mean of the difference between PD after and before the induction in each trial. Severe outliers in PDD were marked and winsorized prior to data analysis. The average of the scores of Q1 and Q3 was used as a measure of subjective ownership of the rubber hand. The average of Q2 and Q4 was used as a control measure as these questions do not specifically relate to ownership. To assess whether taVNS (active vs. sham) and the timing of the cardio‐visual feedback and tactile‐visual feedback modulated the experience of body ownership, we performed two repeated measures analyses of variance (ANOVAs) on PDD, subjective ownership, and the control measure, with within‐subject factors stimulation (active vs. sham), trial type (tactile vs. cardiac). and congruency condition (synchronous vs. asynchronous).

As exploratory analysis, we further investigated whether any observed effects were modulated by individual differences in interoception and by any observed physiological changes. We first investigated whether we could replicate previous findings that greater interoception is linked to higher susceptibility to the RHI by looking at the correlation between scores in the HCT and HDT with PDD and subjective ownership of tactile and cardiac trials during sham stimulation. We calculated correlations between potential moderators of the taVNS effect (namely, scores in the HCT and HDT and the observed change in HR) and the sham‐controlled taVNS induced change in PDD and subjective ownership (calculated as the differences between active taVNS PDD and sham taVNS PDD, and between active taVNS subjective ownership and sham taVNS subjective ownership).

## 3 Results

### 3.1 Stimulation‐Induced Changes in Physiological Indices

Mean values and standard deviations for HR, HRV (RMSSD), and SKNA at baseline and during active and sham stimulation are reported in Table 2. Correlations between measures are reported in the Supporting Information.

**TABLE 2:** Mean values and standard deviations for physiological indices.

Time	Stimulation	Heart rate *M* (SD)	aSKNA *M* (SD)	rMSSDT *M* (SD)
Baseline	Sham	73.37 (18.85)	0.98 (1.22)	46.75 (31.00)
	Active	74.45 (19.71)	0.80 (0.90)	47.00 (30.50)
Stimulation	Sham	72.77 (19.41)	1.17 (0.24)	49.81 (36.21)
	Active	71.48 (19.54)	1.04 (1.06)	52.35 (47.83)

*Note:* Heart rate (HR), skin sympathetic nervous activity (SKNA) and root mean square of successive differences (RMSSD) as a measure of heart rate variability (HRV) as recorded via neuECG and processed by Labchart8 software.

**FIGURE 6:**
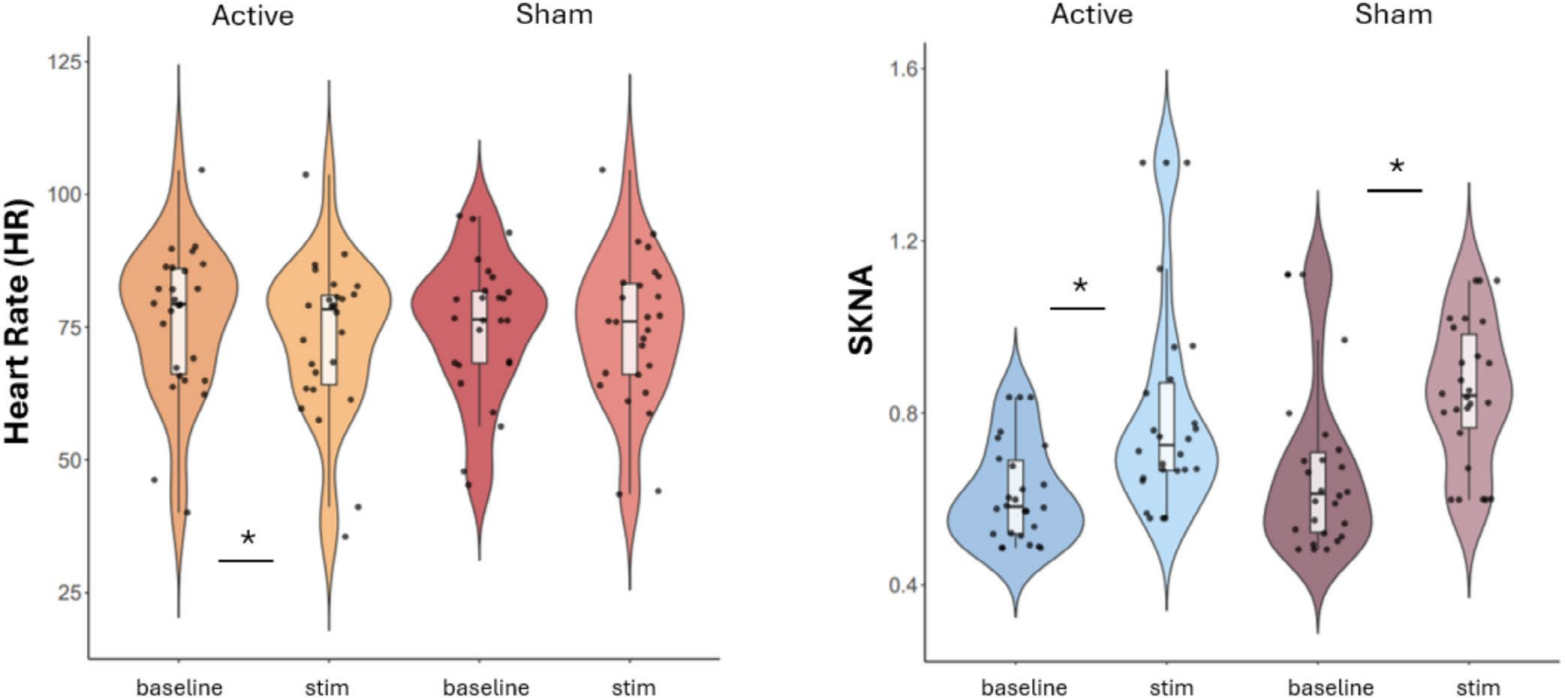
Violin plots displaying significant differences in HR and SKNA between baseline and (active and sham) taVNS. Our findings suggest that HR was lower compared to baseline for active but not for sham stimulation, whereas SKNA increased with the stimulation both during active and sham conditions.

Mean HR was lower during stimulation than baseline during active (*t*(26) = 4.30, *p*_bonf_ < 0.001) but not during sham stimulation (*p*_bonf_ = 1.00). These results suggest that active taVNS may affect sympathetic activity by decreasing heart rate. SKNA data presented severe outliers. With windzorization (0.10), mean SKNA was greater during stimulation than baseline for both sham (*t*(26) = −4.40, *p*_bonf_ < 0.001) and active stimulation (*t*(26) = −4.85, *p*_bonf_ < 0.002), indicating that both types of stimulation may have increased SKNA, potentially because of arousal due to irritation pain, and so forth. Mean RMSSD was not significantly different between baseline and sham stimulation (*p* = 1.00) or between baseline and active stimulation (*p* = 1.00). Significant differences in physiological indices following taVNS stimulation are displayed in Figure 6.

### 3.2 Effects of taVNS and Synchronicity on Ownership of the Hand in the Virtual RHI

Means and standard deviations for scores in PDD and subjective ownership ratings in all conditions of the RHI are reported in Table 3.

**TABLE 3:** Scores for proprioceptive drift difference (PDD) and subjective ownership ratings for all trials of the virtual RHI.

Congruency	Stimulation	Trial type	PDD *M* (SD)	Subjective ownership *M* (SD)
Async	Active	Cardiac	19 (37)	4.704 (1.728)
		Tactile	03 (47)	4.296 (1.898)
	Sham	Cardiac	−06 (45)	4.741 (1.430)
		Tactile	09 (44)	4.222 (1.577)
Sync	Active	Cardiac	05 (42)	4.593 (1.866)
		Tactile	31 (48)	4.630 (1.801)
	Sham	Cardiac	25 (42)	4.815 (1.331)
		Tactile	38 (38)	5.296 (1.325)

*Note:* PDD values reported in cm.

Abbreviations: *M*, mean; SD, standard deviation.

#### 3.2.1 Effects on PDD

**FIGURE 7:**
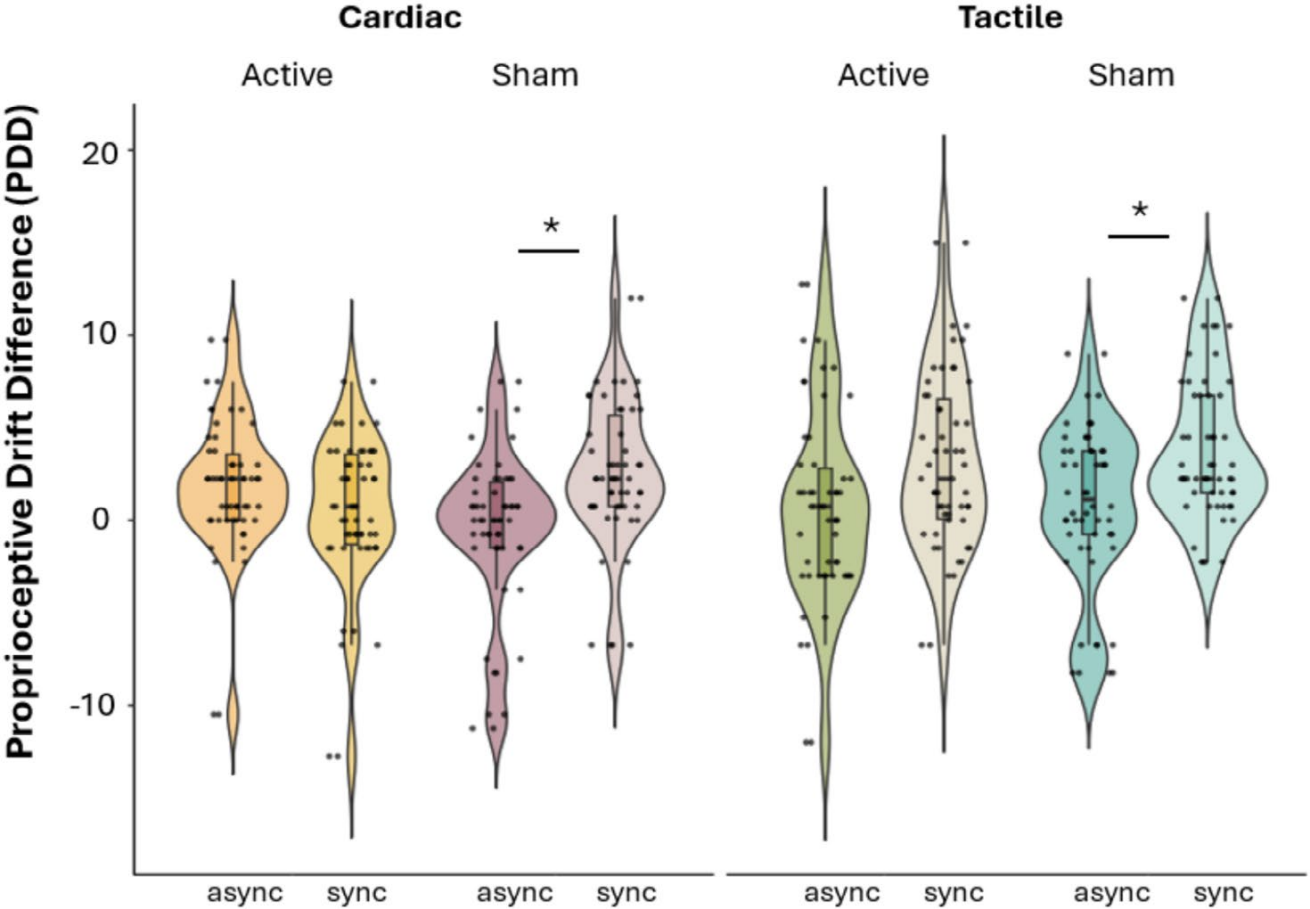
Violin plots displaying significant differences in PDD between active and sham taVNS. In both cardiac and tactile trials, the stimulation seemed to affect susceptibility to the rubber hand illusion. PDD (in cm) was greater for synchronous trials compared to asynchronous trials only during sham stimulation but not during active stimulation.

Violin plots displaying significant differences in PDD between baseline and stimulation for active and sham taVNS are displayed in Figure 7.

The results of the repeated measures ANOVA show a significant effect of synchronicity (*F*(1,26) = 12.97, *p* = 0.001) on PDD, which was higher for synchronous compared to asynchronous trials (*t*(26) = −3.60, *p*_bonf_ = 0.1). No significant main effect of stimulation (*p* = 0.848) or trial type (*p* = 0.060) was revealed; however, a significant interaction of synchronicity*stimulation was found (*F*(1,26) = 9.11, *p* = 0.006). Post hoc tests revealed that synchronous trials presented greater PPD than asynchronous only during sham (*t*(26) = −4.58, *p*_bonf_ < 0.001) but not during active (*p*_bonf_ = 1.00) conditions. These results indicate that, when ignoring trial type, taVNS stimulation may lead to a decreased sensitivity to the RHI. Moreover, a significant effect of synchronicity*trial type was found (*F*(1,26) = 6.19, *p*_bonf_ = 0.020). In the tactile condition, synchronous trials led to greater PDD than asynchronous trials (*t*(26) = −4.28, *p*_bonf_ = 0.001), while in the cardiac condition, synchronous trials did not show greater PDD than asynchronous (*p*_bonf_ = 1.00). These results indicate that, not considering the stimulation, only the tactile trials appeared to elicit the RHI. There was a significant effect of synchronicity*stimulation*trial type (*F*(1,26) = 6.09, *p*_bonf_ = 0.020). Planned contrasts revealed that for tactile trials, synchronous trials led to greater PDD than asynchronous during sham (*t* = −3.98, *p*_bonf_ = 0.003) but not during active stimulation (*p*_bonf_ = 0.102). For cardiac trials, synchronous trials led to greater PDD than asynchronous during sham (*t*(26) = −3.125, *p*_bonf_ = 0.026) but not during active stimulation (*p*_bonf_ = 0.247). These results indicate that the stimulation might have decreased the effectiveness of the virtual induction in inducing the RHI for both cardiac and tactile trials.

#### 3.2.2 Effects on Subjective Ownership

**FIGURE 8:**
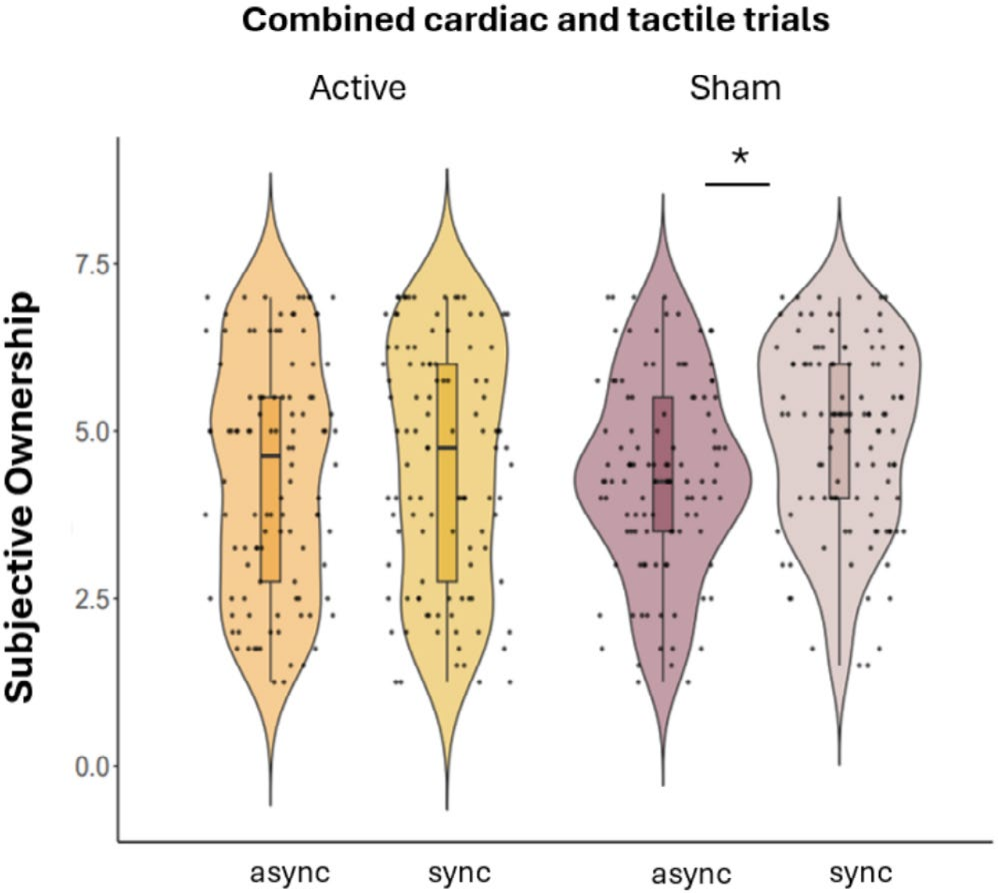
Violin plots displaying significant differences in subjective ownership between active and sham taVNS. In both cardiac and tactile trials, subjective ownership was greater for synchronous trials compared to asynchronous trials only during sham stimulation but not during active stimulation.

Violin plots displaying significant differences in subjective ownership between baseline and stimulation for active and sham taVNS are displayed in Figure 8.

The results of the repeated measures ANOVA revealed a significant main effect of synchronicity (*F*(1,26) = 9.25, *p*_bonf_ = 0.005) on subjective ownership, which was higher for synchronous compared to asynchronous trials (*t*(26) = −3.04, *p*_bonf_ = 0.005), in line with previous research and our findings on PDD. There was no significant main effect of stimulation (*p*_bonf_ = 0.291) or trial type (*p*_bonf_ = 0.627). There was a significant effect of synchronicity*stimulation (*F*(1,26) = 9.18, *p*_bonf_ = 0.005). Post hoc tests revealed that synchronous trials presented greater subjective ownership than asynchronous trials only during sham (*t*(26) = −3.52, *p*_bonf_ = 0.010) but not during active (*p*_bonf_ = 1.00) conditions. These results suggest that, when excluding trial type, taVNS stimulation may have led to a decreased sensitivity to the RHI, both implicitly (as highlighted by our results on PDD) and explicitly. There was a significant effect of synchronicity*trial type (*F*(1,26) = 9.77, *p*_bonf_ = 0.004). In the tactile condition synchronous trials led to greater subjective ownership than asynchronous trials (*t*(26) = −3.59, *p*_bonf_ = 0.008) but in the cardiac condition, synchronous trials did not show greater subjective ownership compared to asynchronous trials (*p*_bonf_ = 1.00). These results suggest that, not accounting for stimulation, only the tactile trials appeared to elicit the RHI also at the subjective level. There was no significant effect of trial type*synchronicity*stimulation (*p*_bonf_ = 0.240).

#### 3.2.3 Effects on the Control Measure

The results of the repeated measures ANOVA on control questions of subjective ownership found no significant results for synchronicity (*p*_bonf_ = 0.957), stimulation (*p*_bonf_ = 0.128), trial type (*p*_bonf_ = 0.081), synchronicity*stimulation (*p*_bonf_ = 0.414), synchronicity*trial type (*p*_bonf_ = 0.478), stimulation*trial type (*p*_bonf_ = 0.373) or synchronicity*stimulation*trial type (*p*_bonf_ = 0.720).

### 3.3 Exploratory Analysis: Investigating if Stimulation Effects Are Moderated by Stimulation‐Induced Changes in Heart Rate and Baseline Interoception

Correlation analysis suggested there was no significant correlation between interoceptive scores in the HCT and HDT with proprioceptive drift difference or subjective ownership for cardiac and tactile trials in the sham condition (all values of *p* > 0.05), so we were unable to replicate findings by Tsakiris et al. (2011) that greater interoception is tied to increased susceptibility to the RHI.

Correlation analysis indicated only a significant correlation between HDT scores and the change in subjective ownership for cardiac synchronous trials (*r* = −0.436, *p* = 0.026) which, however, became non‐significant following Bonferroni correction for multiple comparisons. All other correlations between HCT scores, HDT scores, and HR change scores with the observed difference in PDD and subjective ownership between sham and active stimulation were non‐significant (*p* > 0.05). The complete correlations are presented in the Supporting Information.

## 4 Discussion

The main objective of this study was to investigate whether taVNS could modulate the experience of body ownership in a virtual version of the RHI. We recruited 27 participants, as suggested by our power calculation based on the effect size of a previous similar study (Suzuki et al. 2013). Broadly, our results suggest that taVNS can indeed affect the perception of body ownership, as evidenced by changes in proprioceptive drift difference and subjective ownership ratings, and changes in physiological arousal. Specifically, we found that active taVNS decreased sensitivity to the RHI compared to sham stimulation, suggesting that the procedure may have made individuals more attuned to their real bodily signals and less susceptible to the illusion.

### 4.1 Stimulation‐Induced Changes in Physiological Indices

Our results also suggest that active (compared to sham) stimulation may have affected participants’ sympathetic system, as HR was lower during stimulation compared to baseline for active but not for sham stimulation. This is in line with previous research highlighting physiological changes following taVNS (Antonino et al. 2017; Badran, Mithoefer, et al. 2018; Clancy et al. 2014) and suggests that active taVNS may increase parasympathetic activity. However, the absence of changes in HRV over the 5‐min measurement period implies that while active taVNS effectively decreases HR, it did not produce immediate changes in the broader autonomic balance or variability in heart rate, which typically reflect longer term autonomic regulation and may require longer measurement periods to detect changes (De Couck et al. 2017). Previous research shows inconsistencies in findings related to HRV and taVNS (Burger et al. 2020; Kaniusas et al. 2019; Wolf et al. 2021). The fact that both active and sham stimulation led to an increase in mean SKNA could reflect a sympathetic response to the sensory aspect of the electrical stimulation, which may have caused a degree of discomfort and elevated SKNA. Keute et al. (2018) address criticism against using the earlobe as a sham‐control site for taVNS due to its historical use in acupuncture and the activation of somatosensory and insular areas following earlobe stimulation (Rangon 2018) but the authors suggest that using the scapha as a sham site might reduce sensory differences between active and sham conditions, and suggest usi repeated measures to better control for the effects of sham stimulation. However, studies using earlobe stimulation show no effects related to tVNS suggesting the earlobe can be used as sham (Frangos et al. 2015; Sclocco et al. 2020; Teckentrup et al. 2021; Yakunina et al. 2017).

### 4.2 Stimulation‐Induced Changes in the RHI

Our findings suggest that taVNS can modulate the experience of body ownership by interfering with the processing of body signals. For both tactile and cardiac conditions, during sham stimulation there was a significant effect of synchronicity on PDD and subjective ownership ratings, suggesting that both objective (PDD) and subjective (questionnaire) ownership were higher when visuo‐tactile feedback was synchronous compared to asynchronous. This result mirrors the majority of research on the RHI and replicates the findings from the previous version of this task (Suzuki et al. 2013) in which congruency of tactile and cardiac feedback significantly modulated subjective ownership (see Quadt et al. 2018 for a review). However, during active stimulation, there was no difference in PDD or subjective ownership, suggesting that the stimulation interfered with the effectiveness of the RHI. Our findings extend the evidence by Villani et al. (2019) who demonstrated that taVNS can improve interoceptive accuracy in the heartbeat discrimination task. Unlike Villani et al. (2019), we focused on the modulation of body ownership through the RHI, providing novel insights into the effects of taVNS on higher order self‐processing. These findings are in line with evidence that people with stronger cardiac interoception may be less prone to the illusion (Tsakiris et al. 2011, but see also Crucianelli et al. 2018, Horváth et al. 2020) and that more reliable proprioceptive signals might interfere with the easy integration of visuo‐tactile signals that lead to inferring limb ownership (Chancel et al. 2022; Chancel and Ehrsson 2023). As we observed effects for both cardiac and tactile conditions, it is likely that taVNS effects on body ownership are not due specifically to the processing of cardiac signals, but rather to the general multisensory integration of visual, tactile, and proprioceptive signals that determines the experience of body ownership in the RHI. For instance, Moffatt et al. (2024) demonstrated that while visuo‐tactile synchrony is critical for inducing the RHI, cardiac timing alone does not significantly modulate the illusion. Nevertheless, exploratory findings indicated that individuals with higher interoceptive accuracy showed reduced embodiment during systole compared to diastole, specifically in synchronous conditions. This suggests that interoceptive signals, such as cardiac timing, may exert a subtle modulatory influence on body ownership, contingent on individual differences in interoceptive accuracy.

The mechanisms through which taVNS influences body ownership likely involve complex interactions between neural and physiological processes. The vagus nerve, which conveys visceral signals to the brain (specifically to the NTS, where they are then projected to higher order brain structures) plays a crucial role in maintaining homeostasis and regulating emotions and self‐awareness (Craig 2009; Critchley and Harrison 2013). By stimulating the auricular branch of the vagus nerve, taVNS may enhance the multisensory integration of interoceptive and proprioceptive signals, leading to increased awareness of bodily states and body position, and reduced susceptibility to external illusions like the RHI. VNS has been proposed to influence how sensory information is filtered and processed in brain regions such as the thalamus and somatosensory cortex and has been linked to sensory processing, particularly in the context of sensory gating and attention to sensory stimuli (Chancel et al. 2022, eLife; Chancel and Ehrsson 2023, Cortex). If taVNS impacts the processing of tactile, proprioceptive, or interoceptive signals, making them more reliable, it might weaken the illusion by diminishing the relative influence of perceptual priors, as suggested by Chancel et al. (2022) and Chancel and Ehrsson (2023). Considering the important role of physiological signals in cognitive and emotional function, this finding may also help to explain why stimulating the VNS seems to play a role in processes such as fear extinction (Burger et al. 2016 but see also Burger et al. 2017, 2019), emotional recognition (Colzato et al. 2017; Sellaro et al. 2018), and divergent thinking (Colzato et al. 2018). Alternatively, it is also possible that, as VNS has been shown to influence various cognitive, emotional, and perceptual processes, results may reflect general modulatory effects on processes such as attention or memory consolidation, which may be affected by vagal projections to the nucleus of the solitary tract, which connects to multiple cortical and subcortical regions involved in cognition (Aniwattanapong et al. 2022; Hansen et al. 2003; Sun et al. 2017).

### 4.3 Exploratory Analysis: The Moderating Role of Physiological Changes and Baseline Interoception

As exploratory analysis, we also looked at the correlations between potential moderators of the taVNS effect (scores in the HCT and HDT, and the observed change in HR) and the sham‐controlled taVNS induced change in PDD and subjective ownership (the delta between active and sham values). We were unable to replicate the findings by Tsakiris et al. (2011) that greater interoception is linked to more susceptibility to the RHI, as scores in interoceptive tests did not correlate with PDD or subjective ownership for synchronous cardiac and tactile trials in the sham condition RHI. Following Bonferroni corrections for multiple comparisons, there were no significant correlations between potential moderators and the sham‐controlled taVNS induced change in PDD and subjective ownership. In relation to this, Critchley et al. (2021) reported that autonomic measures such as heart rate, heart rate variability, electrodermal activity, and SKNA showed no significant differences between synchronous and asynchronous conditions in the RHI, nor between individuals who experienced the illusion and those who did not. Thus, it is possible that although taVNS might affect autonomic measures, it may also distinctly enhance interoceptive processing and multisensory integration processes, altering ownership towards the virtual hands in the RHI. However, considering the small sample size, our findings are not sufficient to discard the possibility that individual differences in interoception may affect susceptibility to the stimulation, and we suggest future research should increase sample sizes to gain more power to detect significant effects and highlight the potential complex interactions whereby stimulation and synchronicity may influence the RHI through changes in heart rate and interoceptive sensitivity.

### 4.4 Limitations

This study has several limitations that should be considered. Firstly, the sample size of 27 participants, though based on prior power analyses, is relatively small for detecting subtle effects or interactions, particularly given the assumed effect size of *d* = 0.59. This relatively large effect size, compared to those reported in other studies using taVNS, means the study is not well powered to detect small effects or complex interactions. Secondly, the variability in individual physiological responses to taVNS, such as differences in heart rate and SKNA, introduces complexity in interpreting the effects of the stimulation. Notably, there were many outliers in the SKNA data, which required windsorization to mitigate their impact, also considering the small sample size that did not allow us to remove these data points. This suggests that the SKNA measurements might have been influenced by factors unrelated to the experimental manipulation, such as discomfort or minor pain caused by the stimulation electrodes. Thirdly, the use of a sham‐controlled design, while rigorous, does not fully eliminate the potential influence of participant expectations and placebo effects on the results. As there was no control condition without any stimulation, it is possible that the observed effects could be due to the general impact of receiving any form of electrical stimulation, rather than specific effects of taVNS. This is exemplified by changes in SKNA, which may have mitigated susceptibility to the rubber hand illusion across all conditions. Additionally, a possibility is that earlobe stimulation may also have induced vagal effects, leading to changes in SKNA in both conditions, although these effects were likely greater at the active stimulation site. However, research consistently has used earlobe stimulation effectively as a sham site (Frangos et al. 2015; Sclocco et al. 2020; Teckentrup et al. 2021; Yakunina et al. 2017). An international consensus review by Farmer et al. (2021) considers the vast diversity of stimulation parameters employed in tVNS studies, including sham location, and emphasizes the importance of detailed reporting on stimulation parameters and control conditions to facilitate reproducibility and comparability across studies.

Finally, participants completed interoceptive tests before the VNS stimulation, which might have served as an interoceptive induction, potentially influencing the focus on interoceptive cues and susceptibility to the RHI in all conditions. Future studies should consider larger sample sizes, additional control conditions, including a no‐stimulation condition, and improved methods for handling physiological outliers to better isolate the specific impacts of taVNS on interoceptive processing and body ownership.

### 4.5 Conclusive Remarks

In this study, we provide preliminary evidence that taVNS can affect the experience of body ownership in the RHI by interfering with the processing of visuo‐tactile stimuli. The observed decrease in HR during active stimulation further supports the idea that taVNS can modulate autonomic function, potentially enhancing parasympathetic activity. However, the increase in SKNA across all conditions suggests that the stimulation itself, irrespective of its vagal specificity, may induce general arousal or discomfort, which could impact the overall experience of the illusion and decrease sensitivity to the RHI, both implicitly (PDD) and explicitly (subjective ownership/questionnaires). Our findings contribute to the broader theoretical understanding of body ownership, highlighting the role of the vagus nerve in the multisensory integration of different types of body signals (visual, tactile, proprioceptive and interoceptive) that help us form a coherent sense of self. We suggest taVNS as a new experimental approach to studying corporeal awareness and how different types of sensory information (e.g., interoceptive and proprioceptive) are integrated to determine cognitive and emotional function. The potential clinical applications of taVNS are particularly promising for conditions characterized by altered interoception, proprioception, and body ownership, such as eating disorders, anxiety disorders, and depersonalization/derealization disorders (see Khalsa et al. 2018 for a review). By modulating the processing of bodily signals, taVNS could be used as a noninvasive therapeutic tool to enhance body awareness and emotional regulation in these populations. Future research should address the limitations of this study by incorporating larger sample sizes and additional control conditions, and explore the effects of taVNS across different sensory modalities to provide a more comprehensive understanding of its impact on body ownership. Longitudinal studies examining the long‐term effects of taVNS on interoception and self‐processing would also be valuable.

## Author Contributions

**Alisha Vabba:** conceptualization, data curation, formal analysis, investigation, methodology, project administration, software, validation, visualization, writing – original draft. **Keisuke Suzuki:** investigation, methodology, resources, software, writing – review and editing. **Milica Doric:** data curation, formal analysis, investigation, project administration, writing – review and editing. **Tim J. Möller:** methodology, project administration, resources, software, writing – review and editing. **Sarah Garfinkel:** conceptualization, investigation, methodology, supervision, writing – review and editing. **Hugo Critchley:** conceptualization, formal analysis, investigation, methodology, project administration, supervision, writing – review and editing.

## Conflicts of Interest

The authors declare no conflicts of interest.

## Supporting information


Data S1.

Data S2.

## Data Availability

The study preprint and anonymized datasets analyzed in the present study are available in the OSF repository, https://osf.io/umrz4/?view_only=24bccc2697a6438a94b657e7a75dcef1. The scripts used for presenting the experimental stimuli and collecting participants' responses and raw data are available upon request to the corresponding author (alishavabba@gmail.com).
