# Supplementary material for: The Vagus Nerve as a Gateway to Body Ownership: taVNS Reduces Susceptibility to a Virtual Version of the Cardiac and Tactile Rubber Hand Illusion
Source: Psychophysiology. 2025 Mar 17;62(3):e70040. doi: 10.1111/psyp.70040 (PMC11913772; doi:10.1111/psyp.70040)

**Supplementary materials for “The vagus nerve as a gateway to body ownership: taVNS reduces susceptibility to a virtual version of the cardiac and tactile rubber hand illusion (RHI)”**

# Post-taVNS questionnaire

Subject ID: ________

Date: _____________

|  | **How much did you experience** | **1**  Not at all | **2** | **3** | **4** | **5**  Very much |
| --- | --- | --- | --- | --- | --- | --- |
| 1 | Headache |  |  |  |  |  |
| 2 | Neck pain |  |  |  |  |  |
| 3 | Nausea |  |  |  |  |  |
| 4 | Muscle contraction in the face and/or neck |  |  |  |  |  |
| 5 | Stinging sensation under the electrodes |  |  |  |  |  |
| 6 | Burning sensation under the electrodes |  |  |  |  |  |
| 7 | Uncomfortable (generic) feelings |  |  |  |  |  |
| 8 | Other sensations and/or adverse effects. |  |  |  |  |  |

Describe other adverse effects:

# Mean PDD and subjective ownership across conditions

#
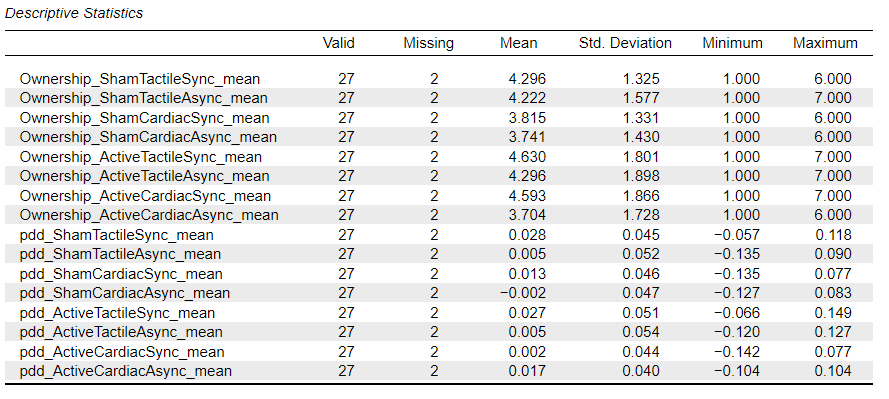


# Mean scores for all survey items across conditions

#
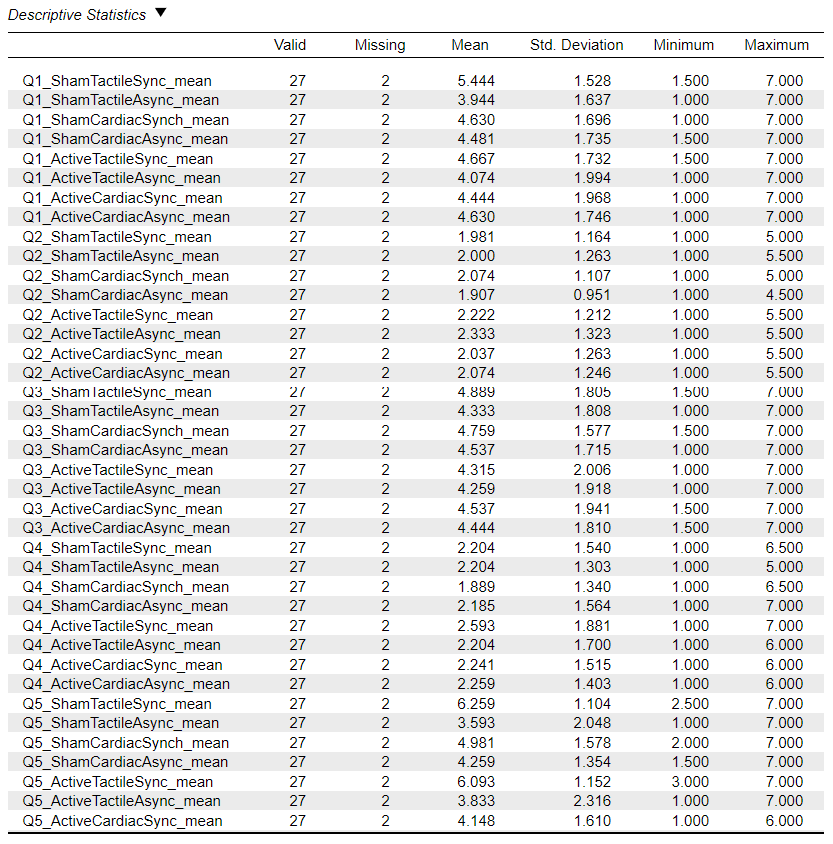


# Correlation matrix for physiological indices


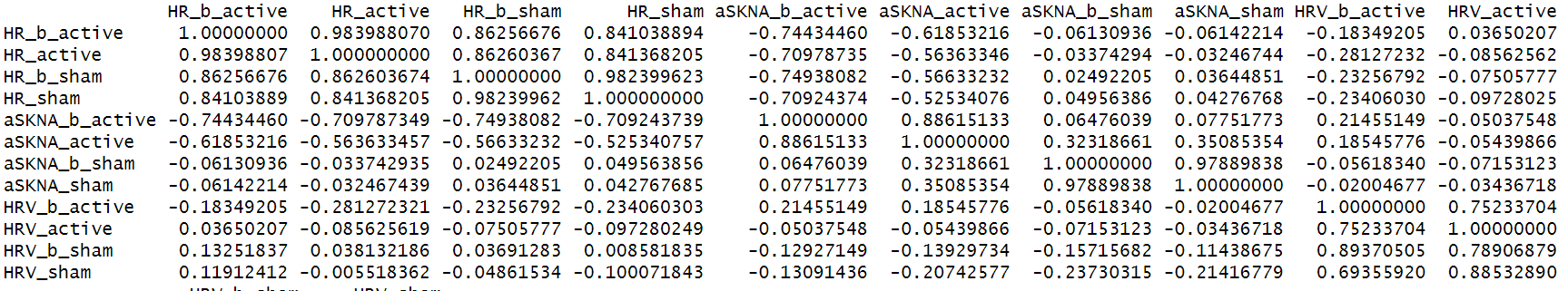


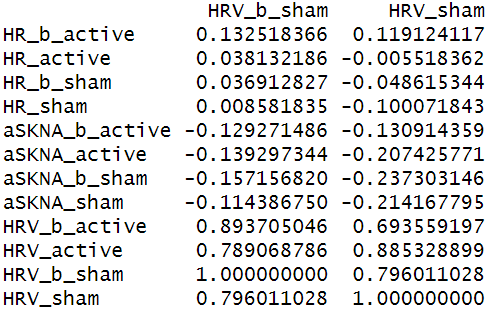

Supplement: Supplementary file 2 — Data S2. [file PSYP-62-e70040-s001.docx]
